# Sex differences in post-stroke aphasia rates are caused by age. A meta-analysis and database query

**DOI:** 10.1371/journal.pone.0209571

**Published:** 2018-12-20

**Authors:** Mikkel Wallentin

**Affiliations:** 1 Department of Linguistics, Cognitive Science and Semiotics, Aarhus University, Aarhus, Denmark; 2 Center of Functionally Integrative Neuroscience, Aarhus University Hospital, Aarhus, Denmark; 3 Interacting Minds Centre, Aarhus University, Aarhus, Denmark; Anadolu University, TURKEY

## Abstract

**Background:**

Studies have suggested that aphasia rates are different in men and women following stroke. One hypothesis says that men have more lateralized language function than women. Given unilateral stroke, this would lead to a prediction of men having higher aphasia rates than women. Another line of observations suggest that women are more severely affected by stroke, which could lead to a higher aphasia rate among women. An additional potential confounding variable could be age, given that women are typically older at the time of stroke.

**Methods & procedures:**

This study consists of two parts. First, a meta-analysis of the available reports of aphasia rates in the two sexes was conducted. A comprehensive literature search yielded 25 studies with sufficient information about both aphasia and gender. These studies included a total of 48,362 stroke patients for which aphasia rates were calculated. Second, data were extracted from an American health database (with 1,967,038 stroke patients), in order to include age and stroke severity into a regression analysis of sex differences in aphasia rates.

**Outcomes & results:**

Both analyses revealed significantly larger aphasia rates in women than in men (1.1–1.14 ratio). This speaks against the idea that men should be more lateralized in their language function. When age and stroke severity were included as covariates, sex failed to explain any aphasia rate sex difference above and beyond that which is explained by age differences at time of stroke.

## Introduction

A stroke is a medical condition in which blood flow to the brain is restricted, due to occlusion (ischemic stroke) or hemorrhage (hemorrhagic stroke), resulting in cell death (WHO). In the US alone, approximately 800,000 people experience a stroke every year, according to the American Heart Association [1]. Stroke is the leading cause of motor and cognitive disability in western countries and aphasia, the inability to comprehend and formulate language because of brain damage, is one of the most common deficits after stroke. A large variability in the reported frequency of aphasia can be found in the literature (e.g. [2–5]), ranging from 15% to 68% of acute patients. A recent meta-analysis, however, concluded that aphasia is present in approximately 30% of acute patients and 34% in rehabilitation settings [6]. Variability of measured rate of aphasia has many causes. The method for aphasia identification differs between hospitals and countries and some sub-scores for aphasia in stroke scales have been found to be limited in their accuracy and reliability [7, 8]. Another potential source of variance may be sex. The above-mentioned meta-analysis did not take potential sex differences into account.

Stroke has been noted to affect the sexes differently. Stroke has been reported to be more common among men [9]. The symptoms of stroke have also been found to differ somewhat between men and women. Women are often more severely affected overall, more often experience paralysis, impaired consciousness and altered mental status together with a generalized weakness, while men more often experience dysarthria, diplopia, sensory loss, ataxia and balancing problems [10]. An association between pre-stroke dementia, which is more prevalent in women, and stroke severity has also been noted [11]. Lastly, aphasia following stroke has been reported to affect women to a larger degree than men (see [10] for a review), although evidence has been conflicting (e.g. [12–14]).

Potential sex differences in aphasia may shed light on overall sex differences in language and cognition. Sex differences in certain linguistic domains are known to exist within the normal population, with differences in first language acquisition speed [15] and reading and writing abilities [16] being the most consistent, favoring girls/women over boys/men. Differences in word use have also been documented [17]. The underlying causes for these differences are probably complex and research trying to tie them to brain structure and function has yielded inconsistent results [18]. Some studies, however, have argued for the hypothesis that language is more bilaterally organized in the brains of women compared to men (e.g. [19–22]), although this is highly controversial [18, 23, 24]. A sex difference in language lateralization would ultimately lead to a sex difference in aphasia following unilateral stroke. If men’s language is more lateralized in the brain than the language of women, we would expect them to be more prone to aphasia following unilateral stroke and vice versa, if women have greater language lateralization than men, we would expect women’s language function to be more vulnerable to stroke.

In this paper I conduct a meta-analysis on aphasia rate given stroke across published peer-reviewed papers and test if the frequency for the two sexes differ. I then compare the results of the meta-analysis to data from a large American patient database (Healthcare Cost and Utilization Project (HCUP) under Agency for Healthcare Research and Quality, U.S. Department of Health & Human Services: https://hcupnet.ahrq.gov). Given that both analyses are based on fully anonymized and publicly available data, the study poses no ethical concerns.

## Meta-analysis methods

This report was prepared according to the Preferred Reporting Items for Systematic Reviews and Meta-Analyses (PRISMA, http://www.prisma-statement.org). PRISMA is an evidence-based minimum set of items for reporting in systematic reviews and meta-analyses [25]. The PRISMA checklist for this meta-analysis is available as supporting information; see S1 File.

**Fig 1:**
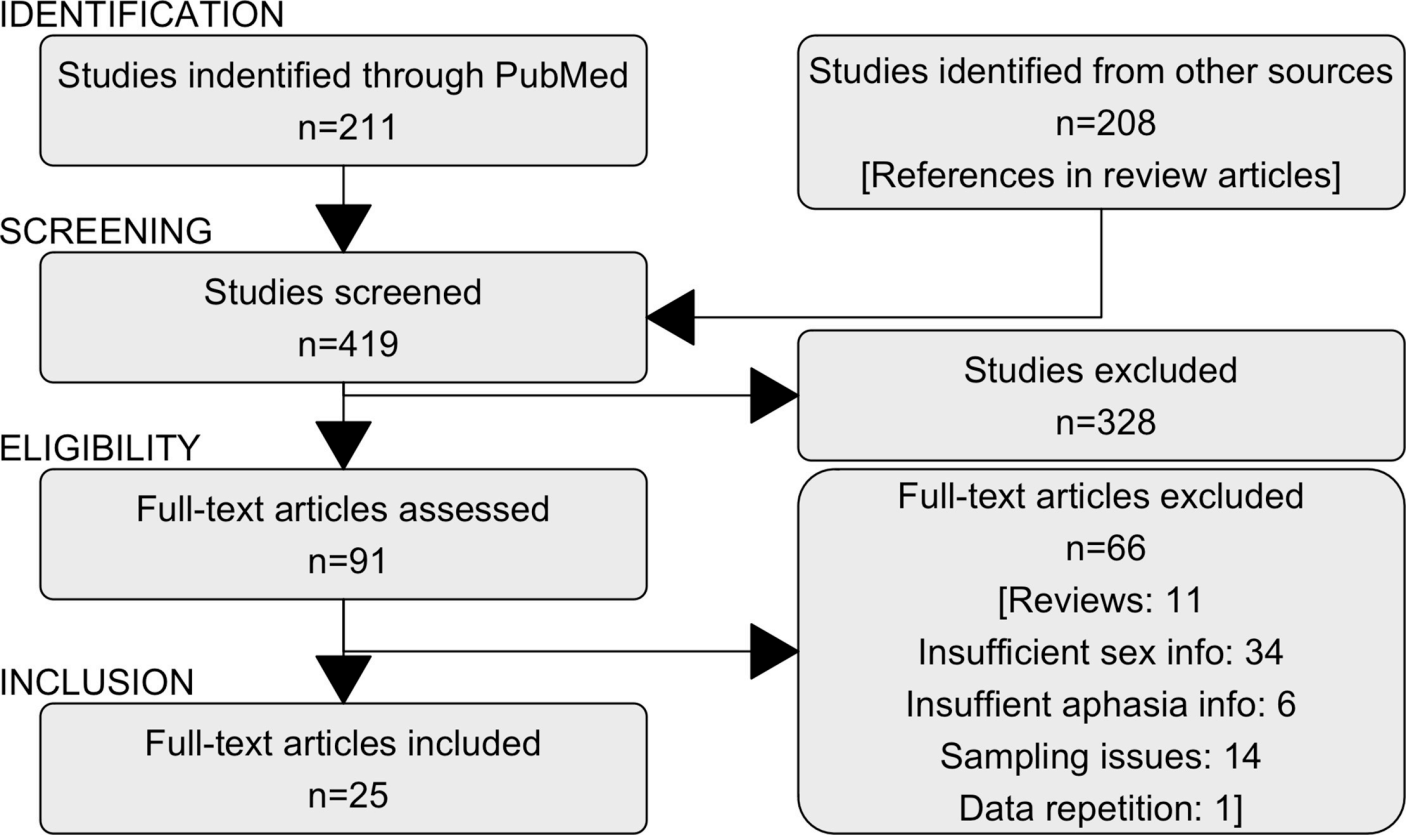
Data gathering flow-chart. Flow chart depicting the different phases of data gathering.

The main outcome measures for the analysis were aphasia rate (percentage of stroke patients with aphasia diagnosis) and the sex ratio of aphasia rates. A pub-med search including the terms “stroke” AND “aphasia” AND “gender” (which automatically includes the term “sex”) generated 211 citations up until July 1st 2018 (see Fig 1 for a flow-chart of the data sampling procedure). No time or language constraints were put on the sampled reports. References in review articles on stroke and aphasia were also investigated. A total of 419 titles were considered. 91 papers were selected for further inspection on the basis of their title and abstract. The analysis set out to study post-stroke aphasia in the broadest sense. Speech pathology tests still lack standardization and diagnostic data for identifying aphasia in stroke populations [26]. Different diagnostic approaches to aphasia were therefore not distinguished. All studies including some overall aphasia diagnosis were deemed relevant. Inclusion criteria were the following: 1) Primary peer reviewed studies reporting aphasia frequency among stroke patients; 2) Studies reporting overall number of aphasia patients and stroke patients from a unselected stroke cohort (thus limiting bias), i.e. studies dealing only with sub-types of aphasia were excluded and studies of group comparisons between aphasia patients and matched control groups were also not considered; 3) Studies reporting aphasia counts for both male and female aphasia patients as well as for stroke patients from both sexes or reports where these numbers could be extracted from reported percentages; 4) First/Primary report of data: The same data could only be included once. If more than one paper investigated the same patient group, the earliest/most comprehensive publication was chosen.

**Fig 2:**
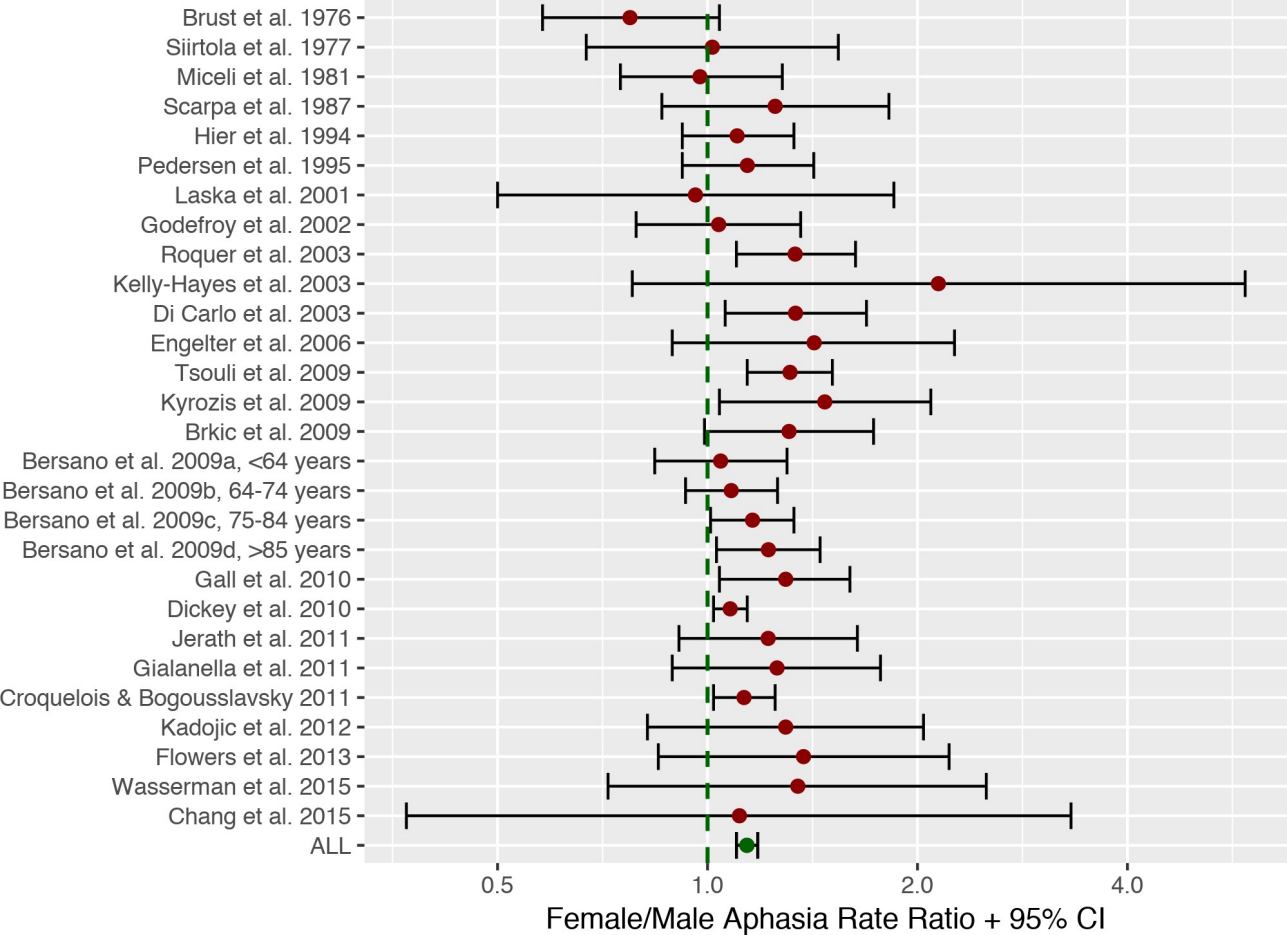
Meta-analysis forest plot. Forest plot of aphasia rate ratios between males and females for the 25 studies included in the meta-analysis (total n = 48,362), showing that across studies a small but significant effect of sex exists, indicating that women are more likely to get aphasia from stroke. This effect, however, does not take age or stroke severity into account.

Twenty-five studies were included in the final dataset [4, 5, 11–14, 27–45]. One study [12] contained information about aphasia frequency for different age bands. These groups were included separately (see Table 1 and Fig 2). I excluded 11 reviews [6, 9, 10, 46–53], 34 studies with insufficient information about the sexes [2, 54–86], 6 studies with insufficient aphasia information [87–92], 14 studies with insufficiently specified sampling procedure for aphasia patients from stroke cohorts [93–106], and one study with repetition of data use [38] (see Fig 1).

**Table 1:** Studies included in the meta-analysis by publication date.

Study	Place	Note	N (stroke)	Aphasia Rate % Female	Aphasia Rate % Male	Ratio	95% CI lower	95% CI upper
Brust et al. 1976 [32]	New York, USA	acute stroke	850	18.4	23.8	0.774	0.580	1.040
Siirtola et al. 1977 [45]	Turku, Finland	acute stroke	338	28.7	28.2	1.016	0.670	1.540
Miceli et al. 1981 [14]	Rome, Italy	223 CVA, 128 tumors, 29 traumas etc.	390	60.8	62.3	0.975	0.750	1.280
Scarpa et al. 1987 [44]	Modena, Italy	left hemisphere stroke, post 14 days	196	62.5	50.0	1.250	0.860	1.820
Hier et al. 1994 [33]	four sites, USA	acute stroke	1805	22.6	19.4	1.103	0.920	1.330
Pedersen et al. 1995 [13]	Copenhagen, Denmark	acute stroke	881	39.7	34.8	1.140	0.920	1.420
Laska et al. 2001 [29]	Danderyd, Sweden	acute stroke	106	33.3	34.7	0.961	0.500	1.850
Godefroy et al. 2002 [5]	Lille, France	Acute stroke, Aphasia	308	68.5	66.1	1.037	0.790	1.360
Di Carlo et al. 2003 [39]	7 European countries	acute stroke	4499	34.8	30.3	1.337	1.060	1.690
Kelly-Hayes et al. 2003 [87]	Framingham MA, USA	at 6 months post stroke	108	23.8	11.6	2.143	0.780	5.900
Roquer et al. 2003 [30]	Barcelona, Spain	acute stroke	1581	28.9	21.6	1.335	1.100	1.630
Engelter et al. 2006 [34]	Basel, Switzerland	acute ischemic stroke	269	34.0	23.9	1.422	0.890	2.260
Kyrozis et al. 2009 [28]	Acadia, Greece	28 days post-stroke	555	27.6	18.8	1.473	1.040	2.090
Tsouli et al. 2009 [4]	Athens, Greece	acute stroke	2297	41.3	31.5	1.313	1.140	1.510
Brkic et al. 2009 [31]	Tuzla, Bosnia and Herzegovina	acute stroke	993	23.0	17.6	1.309	0.990	1.730
Bersano et al. 2009a [12], <64 years	seven regions, Italy	acute stroke, <64 years	1751	21.0	20.0	1.044	0.840	1.300
Bersano et al. 2009b [12], 64–74 years	seven regions, Italy	acute stroke, 64–74 years	2663	26.0	24.0	1.081	0.930	1.260
Bersano et al. 2009c [12], 75–84 years	seven regions, Italy	acute stroke, 75–84 years	2853	31.0	27.0	1.160	1.010	1.330
Bersano et al. 2009d [12], >84 years	seven regions, Italy	acute stroke, 84< years	1581	43.0	35.0	1.223	1.030	1.450
Dickey et al. 2010 [36]	Ontario, Canada	at discharge	15327	33.4	31.0	1.078	1.020	1.140
Gall et al. 2010 [11]	Melbourne, Australia	acute stroke, Dysphasia	843	46.1	35.6	1.294	1.040	1.600
Gialanella et al. 2011 [38]	Lumezzane, Italy	acute stroke	262	55.9	44.4	1.258	0.890	1.770
Croquelois & Bogousslavsky 2011 [27]	Lausanne, Switzerland	acute stroke	5880	28.1	24.9	1.128	1.020	1.250
Jerath et al. 2011 [41]	Rochester, USA	acute stroke	449	45.8	37.4	1.222	0.910	1.640
Kadojic et al. 2012 [37]	Osijek, Croatia	acute ischemic stroke	177	48.2	37.2	1.294	0.820	2.040
Flowers et al. 2013 [35]	Toronto, Canada	acute ischemic stroke	221	35.7	26.0	1.373	0.850	2.220
Wasserman et al. 2015 [42]	Ottawa, Canada	isolated aphasia as only deficit of stroke	1155	4.1	3.0	1.347	0.720	2.510
Chang et al. 2015 [43]	Colombo, Sri Lanka	data from questionnaire	24	62.5	56.2	1.111	0.370	3.320
**ALL**			**48362**	**29.6**	**26.0**	**1.139**	**1.100**	**1.180**

For every study, the number of stroke patients for each sex was extracted together with the number of aphasia patients for each sex and the percentage of aphasia patients. Studies with more patients were considered less at risk of within-study bias and analyses therefore incorporated study size weights. Study year, and place were also noted, as well as potential additional information about the patients’ age and time/type of study relative to disease onset. All data were analyzed using R. Heterogeneity between studies was assessed using the metaphor package [107] and a funnel plot was used to inspect potential publication/selection bias. Year of study was added as a covariate to a subsequent analysis in order to investigate if sex differences in aphasia diagnoses have been changing over time.

## Results of meta-analysis

**Fig 3:**
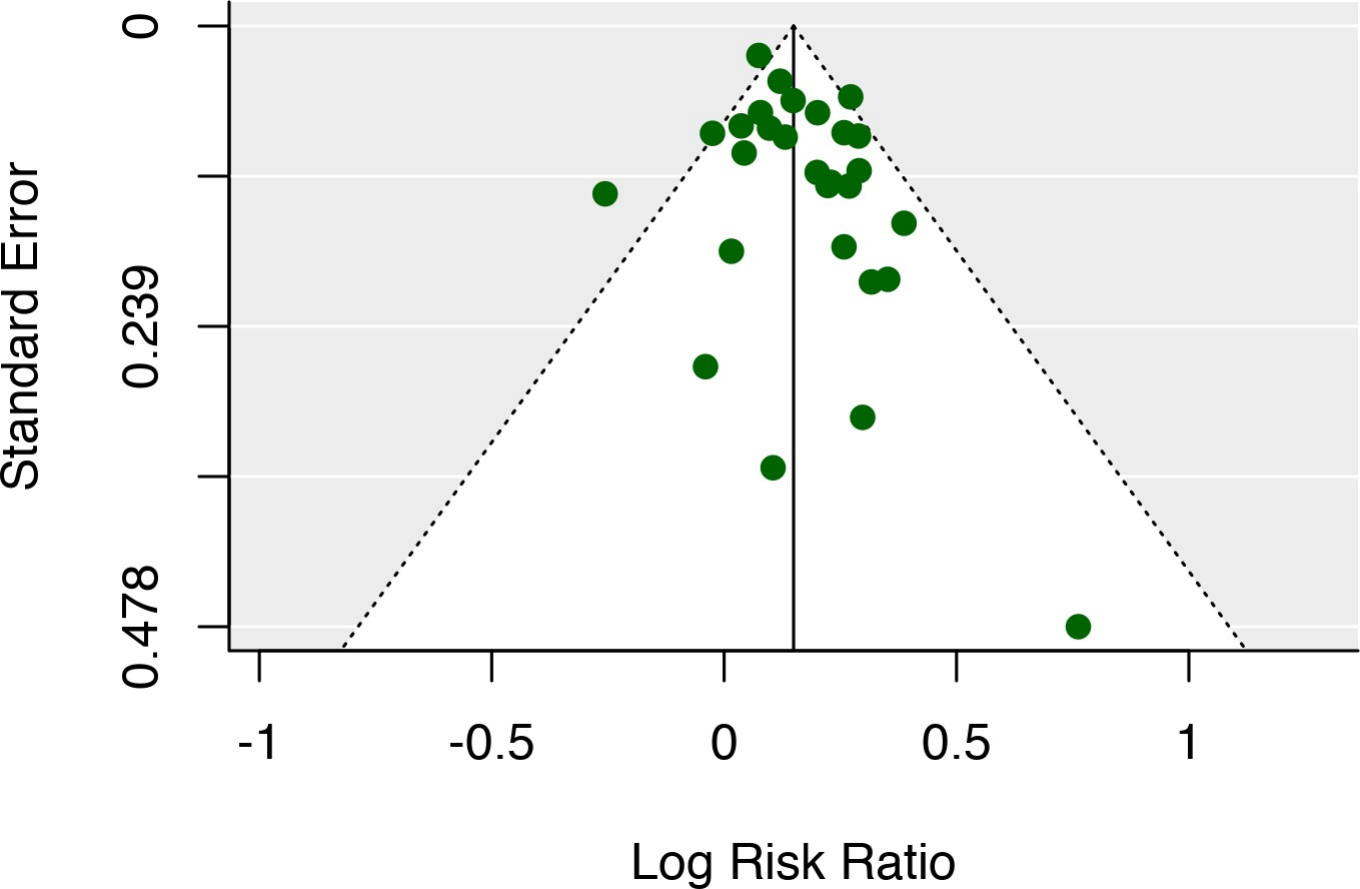
Funnel plot. A Funnel plot did not indicate any outspoken bias in the meta-analysis data.

The 25 studies included a total of 48,362 stroke patients (23,085 women, 25,297 men). Of these 13,398 (6,828 women, 6,570 men) were diagnosed with aphasia (27.7%). 29.6% of female stroke patients were diagnosed, while 26% of males were diagnosed with aphasia (see Table 1 and Fig 2). This difference was found to be statistically significant using a paired and weighted t-test on the aphasia rates across studies, weighted to add emphasis on studies with larger patient samples, *t*(27) = 6.76, *p*<0.001, forcing a rejection of the null-hypothesis that there is no difference in aphasia rate between women and men. The overall sex aphasia rate ratio was found to be 1.14 (1.10–1.18 95% CI) with a Cohen’s *d* of 0.37 which is usually considered a small effect [108]. Low to medium heterogeneity between studies was observed: *I*^2^ = 41% (CI: 4%-77%) (see Fig 1 for a forest plot) and a funnel plot did not suggest any outspoken bias in the reports (see Fig 3). Including publication year as a covariate in a regression analysis did not alter the result and was not in itself a significant predictor of sex differences (p>0.05).

## Interim discussion

A higher aphasia rate after stroke for women than for men was found across studies in the meta-analysis. The average aphasia rate for women was 29.6% and for men 26% (see Table 1). The effect-size was in the small range.

The aphasia rate across studies and sexes (27.7%) was comparable to that reported in a recent meta-analysis (30%) [6]. The slightly lower estimate may in part be related to the inclusion of a study of cases with isolated aphasia [42] which had a much smaller aphasia rate than studies with a regular aphasia diagnosis (see Table 1). The aphasia rate ratio for the sexes reported on isolated aphasia by [42], however, was comparable to that of most other studies in the sample. Study year did not explain any variance in sex differences, suggesting that e.g. evolving diagnostic procedures for aphasia [26] have not had an observable linear impact on sex differences in aphasia rates. The forest plot in Fig 2 is ordered according to publication year on the y-axis, and given that there had been changes as a function of time, this should be visible as either a leftward or rightward trend in the data points. This seems not to be the case.

The fact that a higher aphasia rate after stroke for women than for men was found across studies contradicts the notion that language in men is more lateralized than in women (see introduction). If men have more lateralized language, one would expect their language to be more vulnerable to unilateral stroke than women’s language, which, as shown, is not the case. But the findings are also at odds with previous critical suggestions that there are no sex differences in language lateralization between women and men (e.g. see [18, 109]). At face value, the findings would suggest that women in fact have more lateralized language than men. There are, however, reasons to hesitate before arriving at such a conclusion, based on the present analysis. As mentioned in the introduction, stroke is known to affect men and women differently on a number of accounts, including general severity. The sexes also differ on general health levels, resulting in women being older, on average, when they get a stroke [9]. Age has previously been found to be a predictor of acquiring aphasia [52]. In order to investigate if the effects of sex found in the meta-analysis are specific to language or may relate to more general differences that are unlikely to be caused by a sex difference in core language function, an investigation of aphasia rates that include additional explanatory variables is needed. Unfortunately, very few studies in the current cohort make detailed reports of age effects on aphasia stratified for sex. One exception is Bersano et al. [12] who report aphasia rates for 4 different age groups. Here an interaction between age and sex differences seemingly can be observed. The sex difference is almost non-existing in the youngest age group (under 64), but gradually grows larger and larger in older age groups (see Table 1). It thus seems that taking age into account is important when trying to understand the sex difference in aphasia rates.

Only six studies provided precise gender-stratified age information for their samples (see Table 2). The female stroke groups are older than the male groups in five of these six studies. A post-hoc weighted t-test again found that females were more likely to suffer from aphasia, *t*(5) = 5.1, *p*<0.005, but when adding age difference as a covariate, this effect was no longer significant, *t*(4) = 0.8, *p*>0.05. It could thus seem that age explains some of the difference found between the sexes. The low number of degrees of freedom and the diverse study characteristics, however, make it very difficult to conclude anything definite on the basis of this analysis.

**Table 2:** Studies in the meta-analysis with age information.

Study	N (stroke)	Aphasia Rate % Female	Aphasia Rate % Male	Ratio	Age Female	Age Male	Age difference
Hier et al. 1994 [33]	1805	22.6	19.4	1.165	69.2	65.3	3.9
Kelly-Hayes et al. 2003 [87]	108	23.8	11.6	2.052	80.3	75.8	4.5
Roquer et al. 2003 [30]	1581	28.9	21.6	1.335	74.6	68.8	5.8
Gall et al. 2010 [11]	843	46.1	35.6	1.294	76.0	72.0	4.0
Jerath et al. 2011 [41]	449	45.8	37.4	1.225	79.0	70.0	9.0
Chang et al. 2015 [43]	24	62.5	56.2	1.111	61.6	64.7	-3.1

Another possible explanation for the higher aphasia rate in women is that they may be affected more severely by stroke in a non-discriminant manner [10]. If aphasia rates can be explained by severity alone, it would again suggest that the sex difference is not restricted or related to language in any meaningful way. But again, this type of information is not reported in the papers covered by the present meta-analysis.

An additional limitation in the present meta-analysis is that the literature has been surveyed and studies have been selected by a single individual, the author. This carries the potential risk of conscious or nonconscious selection bias. Speaking against this is the fact that the observed sex difference in aphasia goes directly against the conclusions of the author’s prior work. I have argued against the existence of large sex differences in the brain architecture supporting language, both in review articles [18, 110] and based on neuroimaging studies [109, 111]. Before starting the data collection, I assumed that there would be no differences in aphasia related to sex. If anything, a potential selection bias based on the author’s bias, would most likely mean that the observed sex difference is underestimating the real difference. Inspection of the funnel-plot did not reveal bias in the selected studies, and I consider this risk rather small, but it is a possibility.

One way to confront this problem and counter the potential age and severity confounds at the same time is to get access to more detailed aphasia data with additional age and severity information. One suggestion could be to contact the authors of the studies included in the meta-analysis and try to obtain the relevant information. However, given the large time span covered by the included publications, this would be unfeasible. I have therefore instead added a 2^nd^ dataset from an American healthcare database (see below) that will allow me to investigate aphasia rates while taking age and stroke severity into account.

## Methods for database analysis

Data from the Healthcare Cost and Utilization Project (HCUP) from community hospitals in the United States were used for the analysis. The database (https://hcupnet.ahrq.gov/) contains data from the National Inpatient Sample (NIS) using the International Classification of Diseases and Health Related Problems (ICD-9) codes from 35 US American states from the years 2011–2014. The ICD system is used by US hospitals for reimbursement purposes and subsequent research, e.g. to study patterns and outcome of disease [112]. Starting in data year 2012, the NIS is a sample of discharge records from all HCUP-participating hospitals. For prior years (i.e. including 2011), the NIS was a sample of hospitals from which all discharges were retained. Patient counts for each year from each state, stratified by sex, was used in the analysis. Data from this database has previously been used to study post-stroke aphasia rates [2], but here we add sex, age and severity as explanatory variables and incorporate all available states for all the years in which the ICD-9 diagnoses were used (i.e. 10 times more patients).

To identify number of patients with stroke, the combined number of diagnoses from the database related to stroke was obtained. To emulate the studies in the meta-analysis as much as possible, both hemorrhagic and ischemic stroke cases were included in the analysis. Cases with the following ICD-9 codes were thus selected: “431 Intracerebral Hemorrhage“, “434.00 Crbl Thrmbs Wo Infrct”, “434.01 Crbl Thrmbs W Infrct”, “434.10 Crbl Emblsm Wo Infrct”, “434.11 Crbl Emblsm W Infrct”, “434.90 Crbl Art Oc Nos Wo Infrc”, “434.91 Crbl Art Ocl Nos W Infrc”, “436 Cva”. Cases under the ICD-9 codes “433*” (occlusion and stenosis of precerebral arteries) were not included because they have been found to have poor predictive power for acute stroke [113]. To identify the number of patients with aphasia, the ICD-9 code: “784.3 Aphasia” was used. As a proxy for stroke severity, the number of hemiparesis/hemiplegia diagnoses were included (using the ICD-9 code: “342.90 Unsp Hemiplga Unspf Side”, which is the most commonly used ICD-9 code for hemiplegia/paresis in the database and which does not bias towards the dominant or nondominant side). Hemiparesis, hemiplegia and aphasia are comorbid deficits. Around 90% of aphasia patients have hemiparesis [64], but if a sex difference in number of aphasias is accompanied by a similar sex difference in hemiparesis/hemiplegia diagnoses, then the difference is likely to be explained by stroke severity rather than being a specific language related phenomenon.

The database allows for two different ways to draw data. Either one can draw "Principal" diagnoses or “all-listed” diagnoses. As aphasia is often unlikely to be the principal diagnosis in a hospital visit, “all-listed” diagnoses were used. However, age information is only available with “principal” diagnoses, and age information was therefore drawn from this dataset. The assumption is that age differences in principal diagnosis will be representative for age differences in the “all-listed” diagnoses as well. It is important to note that the database does not offer information about age for any individual patient. These data are all summery data for the participating states for a particular year (2011–2014). Yet, similarly to a meta-analysis of data from different studies, summary data from different states, each representing a large group of patients may nevertheless be representative of generalizable differences between groups (males and females) in the data.

To evaluate statistical significance of the predictors, a linear mixed-effects regression analysis was conducted, fit by REML, using the *lmertest* package in R [114]. P-values were estimated using Satterthwaite's method. The model incorporated aphasia rate as the dependent variable and sex as the main fixed dependent variable. Age and rate of hemiplegia diagnoses (proxy for stroke severity) were z-score scaled and added as additional covariates. The model also included all possible interactions between the three variables. US state and year for each data-point were included as random effects. The regression was weighted by number of stroke cases in a particular state/year, to put more weight on data-points from larger states.

## Results of database analysis

**Fig 4:**
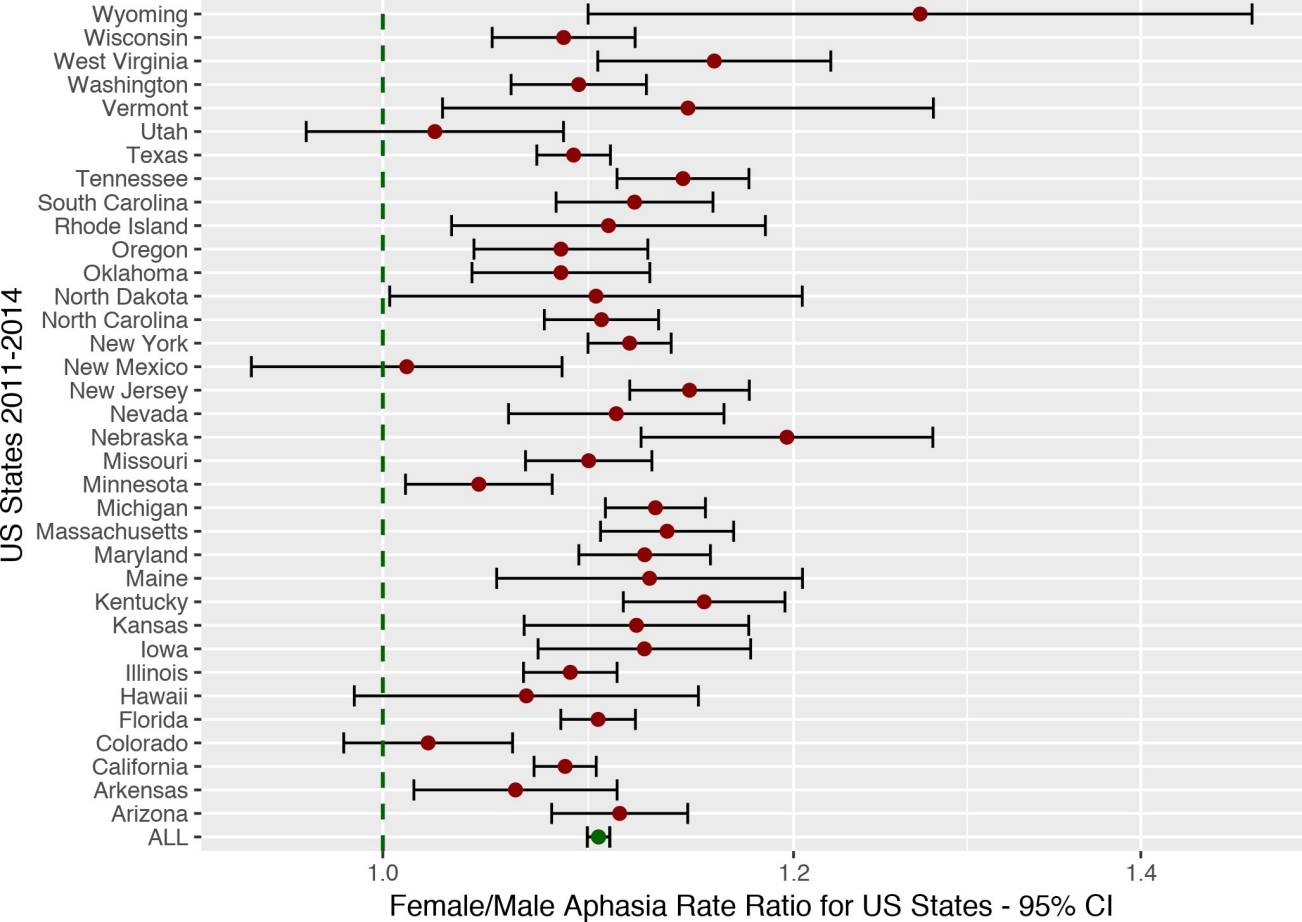
US data forest plot. Aphasia rate ratios (uncorrected for age) for each US state in the HCUP database from 2011–2014. This analysis replicates the findings from the meta-analysis and provides unequivocal evidence for a higher aphasia rate among women compared to men given stroke (see Fig 1, but note the scale difference between plots). However, as Fig 5 shows, this effect can be explained completely by the sex difference in age at stroke.

A total of 1,967,038 stroke patients were found in the database (1,014,239 women, 952,799 men) in the period from 2011 to 2014. Aphasia was diagnosed in 623,942 cases (336,604 women, 287,338 men) or 31.7%. Using this method, 33.2% of female stroke patients were diagnosed, while 30.2% of males were diagnosed with aphasia (see Fig 4).

The overall female/male aphasia rate ratio was found to be 1.100 (1.095–1.106 95% CI) with a Cohen’s *d* effect size across states of 0.63 which is usually considered a medium effect size [108]. A paired t-test again yielded support to the existence of a sex difference, *t*(143) = -18.36, *p*<0.001.

**Fig 5:**
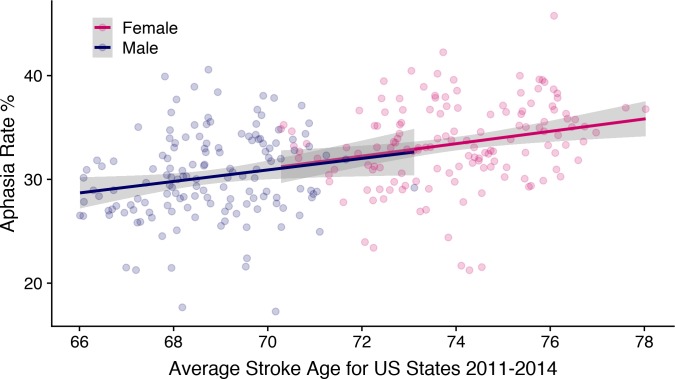
Aphasia rate as a function of age at stroke. A scatterplot of stroke average age against aphasia rate for each US state and year (2011–2014) in the HCUP database. The plot illustrates the large age difference between men and women at time of stroke. It also shows a positive correlation between average age and aphasia rate, suggesting that older stroke patients more often get aphasia. When this relationship is taken into account, sex effects are no longer significant in the aphasia rates.

When including age and stroke severity in a regression analysis, however, no significant effect of sex over and above that explained by age and severity could be observed, *t*(273.11) = -1.64, *p* = 0.1. A significant effect of age, *t*(274.83) = 2.11, *p(uncorrected)*<0.05, and a significant effect of stroke severity, *t*(268.73) = 4.77, *p*<0.001 was observed. Fig 5 displays how sex is completely confounded by age of stroke and does not add any explanatory power to the analysis. A significant interaction between age and stroke severity was also observed, *t*(275.31) = -2.02, *p(uncorrected)*<0.05. No other interactions were significant.

## Discussion

On this very large cohort of patients, we replicate the findings from the meta-analysis. The overall aphasia-rate for the two studies are compatible. The meta-analysis suggested a weighted average aphasia-rate of 27.7%, whereas the aphasia-rate estimated from the database was 31.7%. Both results are comparable to the estimate reported in another recent meta-analysis (30%) [6], where sex was not considered. Both analyses revealed that, based on raw aphasia rates, women are more likely to get an aphasia diagnosis following stroke than men. The sex aphasia rate ratio was slightly smaller in the database study (1.10) than in the meta-analysis (1.14), but the confidence intervals overlap. This speaks against any large selection bias in the meta-analysis. The effect size, as measured by Cohen’s *d*, was found to be larger in the database-analysis (0.63) than in the meta-analysis (0.37). This is likely due to the fact that the database data is much more homogeneous than the data included in the meta-analysis, where the publication time differs substantially (covering the years 1976 to 2015), the time of diagnosis post-stroke differs and the definition of aphasia also differs to some extent (e.g. one study includes dysphasia as well as aphasia and one study only looks at isolated aphasia cases–see Table 1 for details).

At the same time, the 2^nd^ analysis supports the findings from the post-hoc analysis, where age was found to explain away the effect of sex in the subset of papers that had age information. In the database, we find no evidence of any sex difference in aphasia rates over and above that which can be explained by the age differences between females and males at time of stroke. This replicates previous findings that age is a predictor of aphasia following stroke [52], and given that age is a more fundamental causal variable than language (i.e. your language cannot change your age, but the opposite may be true), it is likely that age is the cause of this statistical relationship and also that most, if not all, of the sex differences in aphasia rates are caused by the age difference in stroke between women and men. Fig 5 illustrates this. The regression line for aphasia rate as a function of age for females is an exact continuation of the same regression for males. No offset to imply a main-effect of sex.

I also found an independent effect of stroke severity on aphasia rates as measured by diagnoses of hemiplegia. Aphasia and hemiparesis/hemiplegia are known to be highly co-morbid. In this study I found that severity effects on aphasia are independent of the sex effects. The sex differences thus do not seem to be related to stroke severity per se. It has to be said, however, that this analysis used a somewhat crude proxy for stroke severity. Other measures, such as general stroke scale scores [115, 116] might interact more with sex.

Bersano et al. [12] found indications of increasing sex differences in aphasia rates with age. Contrary to this, the database analysis showed no indication of an interaction between sex and age. Bersano and co-workers did not report inferential statistics documenting an actual interaction, but looking at their data, the increasing discrepancy in aphasia between males and females with age is striking. For patients below 64 years the aphasia rate gender ratio is 1.04 and grows to 1.08 in 64 to 74 year-old patients, 1.16 in 75 to 84 and 1.22 in patients above 84 years of age (see Table 1). How does this fit with the current data not showing any interaction between age and sex? One possible explanation is that there is an inherent bias in the way that the Bersano and co-workers’ age data is distributed. When lumping the data into 10-year age bins, one needs to consider that women and men may not be equally distributed within each bin. The data from the Bersano et al. study were collected in 2001 in Italy. If one looks at the gender and age distribution of the Italian population in January 2002 using population statistics (http://demo.istat.it/pop2002/index_e.html), one finds that because women live longer than men, the average age of women within the different age bins from middle age and onwards is higher than that of men and that this difference gets larger for the older groups. For the 54 to 64 year-old Italians, the mean age difference between men and women is 0.07 years, but for the age group above 84 years, it has grown to 0.52 years. There is a very strong linear correlation between the mean age differences in the Italian population in these age bins and the reported differences in aphasia rate (*r* = 0.96, data available from author on request), which suggests that at least some of the interaction between sex and age seen in the Bersano and co-workers’ data is based on unequal sampling of the different ages. This is not to say that there could not be sex and age interactions that were not picked up by the current analysis. The data from the database is distributed on a state by year basis and each data-point for age is the result of averaging across many individual patients. Underneath this gross data reduction may be hidden lots of important variability. Further studies are needed in order to rule out a potential interaction between sex, age and aphasia.

The present analyses are also limited in that they say nothing about the different types of aphasia symptoms that patients may suffer from and the potential interactions that might be found with gender if one looks more carefully at aphasia subtypes.

Taken together, the results are in line with a critical stance towards any large-scale brain base for sex differences in language [18]. This, of course, does not mean that the observed sex differences in language related behavior (see introduction) do not have brain correlates, just that these differences will be dynamic, complex and to a large extent dependent on gender differences in experience and context rather than being tied to genetic sex.

Aphasia is a strong predictor of clinical outcome in stroke [4, 6]. The clinical implications of the present results are that sex can be used as a weak predictor for aphasia in stroke patients in the absence of knowledge about age. This, of course, will only have limited applicability. Apart from this, the findings are in line with results from stroke treatment studies that fail to find effects of sex on stroke outcome and support a non-gender-biased approach to treatment [117]. Age, on the other hand, is likely to be an important factor in the diagnosis and treatment of aphasia. This points to the need for a better understanding of the relationship between language and ageing, both in the healthy and in the clinical population.

## Conclusion

Women were found to be diagnosed with aphasia following stroke more often than men. This is in direct opposition to the hypothesis that women have less lateralized language function than men. The sex difference was found to most likely be caused by age differences in the two groups at the time of stroke.

## Supporting information

S1 File PRISMA checklist. Checklist for meta-analyses according to http://www.prisma-statement.org.
(PDF)
